# Supplementary material for: Biofilms preserve the transmissibility of a multi-drug resistance plasmid
Source: NPJ Biofilms Microbiomes. 2022 Dec 9;8:95. doi: 10.1038/s41522-022-00357-1 (PMC9732292; doi:10.1038/s41522-022-00357-1)
Supplement: Supplementary file 1 — Supplementary Material [file 41522_2022_357_MOESM1_ESM.pdf]

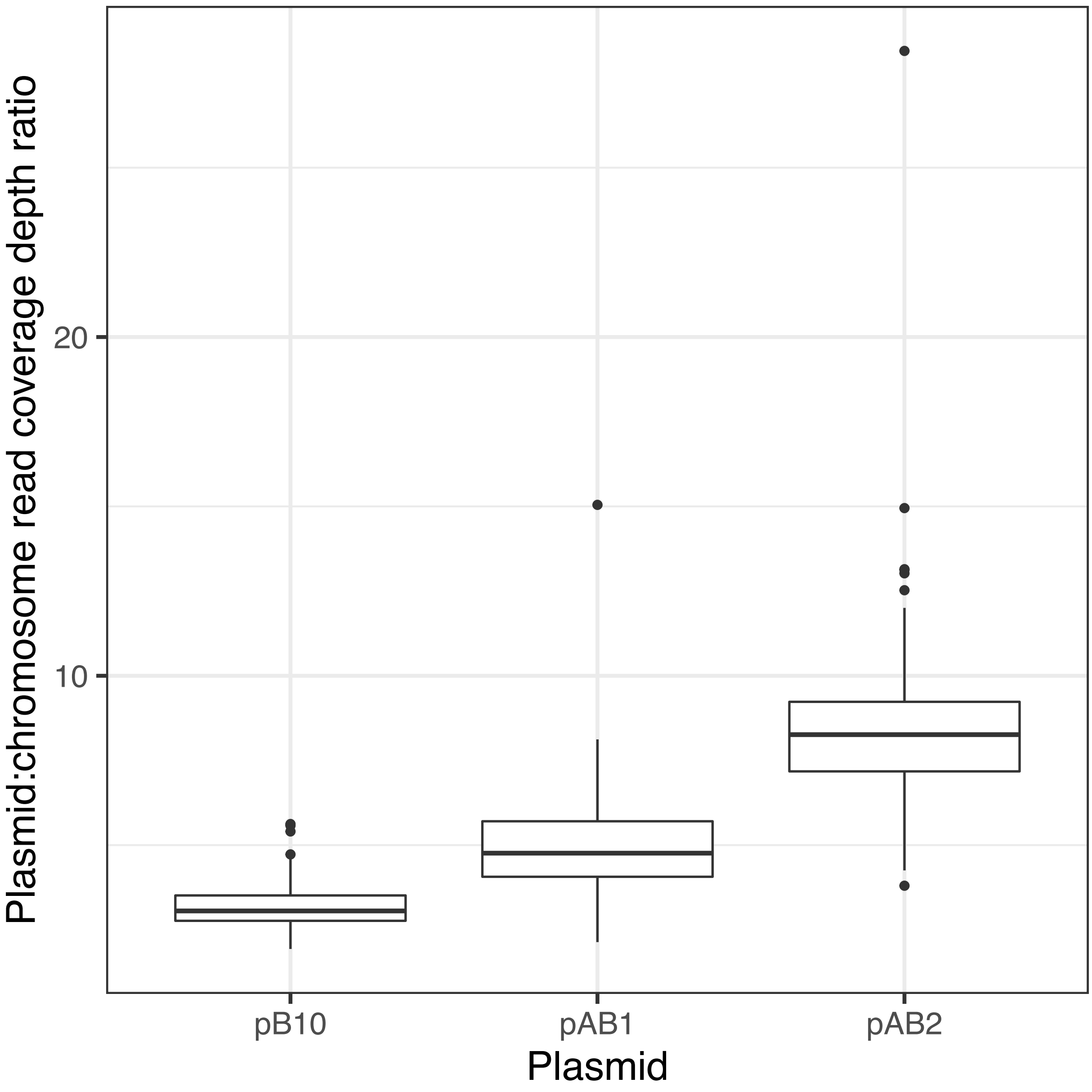

Supplementary Figure 1: Plasmid copy number of the ancestral and all evolved clones based on plasmid-chromosome read coverage for all whole genome sequences.

Supplementary Table 1: Summary of evolved plasmid genotypes with their affected genes and functions, found in all 144 sequenced clones

Antibiotics	Environment	Time Point	Gene(s)	Mutations	Gene Function	Population Replicate	Direct Repeats that Flank Deletions (5'-3'), if present
**Tet+**	Starting Point	t_0	*kfrA*	Δ17 bp	KfrA protein in maintenance/control region	C	
			" "	Δ141 bp	" "	C	
			" "	2 SNPs	" "	C	
			trbL	Δ405 bp	putative DNA topoisomerase in transfer region	C	
	Biofilm	t_{14}	No plasmid mutations at all				
		t_{28}	*trbD–kfrA*	Δ33,164 bp	34 genes in transfer and maintenance/control regions	A	GGCCTG
			trbB–traL	Δ31,764 bp	32 genes in transfer region	A	
			trbE–kfrC	Δ30,220 bp (x2)	31 genes in transfer and maintenance/control regions	C	GGCGGCCTCC
			trbB–kfrC	Δ32,730 bp	34 genes in transfer and maintenance/control regions	C	CGCGGACGGC
			trbJ	Δ9 bp	conjugal transfer protein TrbJ	C	
	Planktonic	t_{14}	*trbF–kfrA*	Δ29,446 bp (x7)	32 genes in transfer and maintenance/control regions	A, B	GCGCGCGC
			xf2080 / tetR [2]	SNP	hypothetical protein / tetracycline resistance repressor	A	
			trbE–traM	Δ28,988 bp (x5)	30 genes in transfer region	B	GTGGCCGCCG
			trbE–kfrA	Δ30,679 bp (x6)	33 genes in transfer and maintenance/control regions	C	
		t_{28}	*trbE–traM* [3]	Δ28,549 bp (x6)	30 genes in transfer region	A	GGCCAaCTC*
			kfrA [2]	Δ84 bp	KfrA protein in maintenance/control region	A	
			trbE–traM	Δ28,988 bp (x6)	30 genes in transfer region	B	GTGGCCGCCG
			trbE–kfrA	Δ30,679 bp (x6)	33 genes in transfer and maintenance/control regions	C	
**Tet-**	Starting point	t_0	*klcB*	Δ144 bp	KlcB protein in maintenance/control region	A	
	Biofilm	t_{14}	*trbL*	Δ219 bp	putative DNA topoisomerase in transfer region	A	
			trbL	Δ306 bp	putative DNA topoisomerase	B	
			trbB	SNP	conjugal transfer protein TrbB in transfer region	B	
			trbI	Δ648 bp	conjugal transfer protein TrbI in transfer region	B	
			trbL–trbN	Δ1,881 bp (x2)	3 genes in transfer region	A	
			trbB–traM	Δ31,451 bp	33 genes in transfer region	C	CGCGGCGC
			trbB–traC	Δ21,755 bp	23 genes in transfer region	C	CCTGGTCGGCGA
	Planktonic	t_6 [4]	*trbE–incC2*	Δ34,594 bp (x4)	36 genes in transfer and maintenance/control regions	B	GGCCGCCTGG
		t_{10} [4]	*trbA–traD*	Δ23,369 bp (x4)	25 genes in transfer region	C	
			trbE–kfrA	Δ31,221 bp (x2)	33 genes in in transfer and maintenance/control regions	C	
		t_{14}	*trbC–kfrA*	Δ32,964 bp(x6)	35 genes in transfer and maintenance/control regions	A	CGGTGGCCTTC

[1] The numbers in parentheses (x2, x4, x5, x6, x7) refer to the number of clones that showed that exact genetic change. When two genetic changes are shown for the same plasmid gene or region at a particular time point, these were observed in separate clones.

[2] These mutations were found in one clone that also has the mutation listed right above

[3] A plasmid with this genotype was tested in other bacterial species (Fig. 5).

[4] Plasmid-containing clones were no longer detected after Day 6 (or 10), explaining why clones were sequenced at these time points in planktonic populations without tetracycline

* Direct repeat right had a 'G' instead of an 'A'

Supplementary Table 2: Matings with various *A. baumanni* clones as donor and *E. coli* as recipient show that loss of transfer genes disabled conjugative plasmid transfer

Donor	Recipient	D (cfu/ml) [a]	R (cfu/ml) [a]	T (cfu/ml) [a]	T/R	Standard deviation	95% CI	T/D	Standard deviation	95% CI
Ancestor	*E. coli* K12Nal[b]	2.7E+09	6.8E+07	3.4E+03	5.3E-05	2.9E-05	7.3E-05	1.3E-06	6.3E-07	1.6E-06
Evolved clone with intact plasmid	" "	9.7E+08	6.5E+07	3.4E+02	5.4E-06	1.7E-06	4.2E-06	3.6E-07	1.2E-07	2.9E-07
Evolved clone with truncated plasmid	" "	2.0E+09	6.4E+07	<10	<1.6E-07	-	-	-	-	-

[a] Values are averages from three replicate mating assays

[b] E. coli K12 Nal is a nalidixic acid resitant mutant of MG1655

Supplementary Table 3: List of all sequenced strains with their predicted mutations generated by Breseq using the ‘gdtools COMPARE’ subcommand.

Predicted mutations

seq id	position	mutation	annotation	gene	description
AJ564903	340	Δ23,369 bp		[trbA]–[traD]	**25 genes** Show
AJ564903	566	Δ31,764 bp		trbB–[traL]	**32 genes** Show
AJ564903	771	G→A	M1M (ATG→ATA) †	trbB →	putative ATPase, autophosphorylase
AJ564903	904	Δ32,730 bp		[trbB]–[traN]	**34 genes** Show
AJ564903	1,452	Δ21,755 bp		[trbB]–[traC]	**23 genes** Show
AJ564903	1,669	Δ31,451 bp		[trbB]–[traM]	**33 genes** Show
AJ564903	1,937	Δ32,964 bp		[trbC]–[kfrA]	**35 genes** Show
AJ564903	2,309	Δ33,164 bp		[trbD]–[kfrA]	**34 genes** Show
AJ564903	2,731	Δ34,594 bp		[trbE]–[incC1]	**36 genes** Show
AJ564903	3,700	Δ30,220 bp		[trbE]–[traN]	**31 genes** Show
AJ564903	3,704	Δ31,221 bp		[trbE]–[kfrA]	**33 genes** Show
AJ564903	4,181	Δ28,988 bp		[trbE]–[traM]	**30 genes** Show
AJ564903	4,301	Δ30,679 bp		[trbE]–[kfrA]	**33 genes** Show
AJ564903	4,731	Δ28,549 bp		[trbE]–traM	**30 genes** Show
AJ564903	5,481	Δ29,446 bp		[trbF]–[kfrA]	**32 genes** Show
AJ564903	7,563	Δ648 bp	coding (290-937/1422 nt)	trbI →	TrbI protein
AJ564903	9,268	$(GCGCAGGGC)_{2\rightarrow1}$	coding (553-561/765 nt)	trbJ →	TrbJ protein
AJ564903	10,666	Δ219 bp	coding (939-1157/1719 nt)	trbL →	putative DNA topoisomerase
AJ564903	10,697	Δ1,881 bp		[trbL]–[trbN]	[trbL], trbM, [trbN]
AJ564903	10,972	Δ405 bp	coding (1245-1649/1719 nt)	trbL →	putative DNA topoisomerase
AJ564903	35,210	Δ84 bp	coding (336-419/1032 nt)	kfrA ←	KfrA protein
AJ564903	35,211	Δ17 bp	coding (402-418/1032 nt)	kfrA ←	KfrA protein
AJ564903	35,225	Δ141 bp	coding (264-404/1032 nt)	kfrA ←	KfrA protein
AJ564903	35,241	G→A	L130L (CTG→TTG)	kfrA ←	KfrA protein
AJ564903	35,338	G→C	A97A (GCC→GCG)	kfrA ←	KfrA protein
AJ564903	40,050	Δ144 bp	coding (579-722/1209 nt)	klcB ←	KlcB protein
AJ564903	46,936	A→G	pseudogene (10638/14051 nt)<br>pseudogene (5865/8895 nt)	tnpA ←<br>tnpA ←	hypothetical protein<br>hypothetical protein
CP000521	17,639	$(TTCTTTGGA)_{11\rightarrow9}$	intergenic (+247/+1487)	A1S_0014 → / ← A1S_3477	tyrosyl-tRNA synthetase/hypothetical protein
CP000521	17,648	$(TTCTTTGGA)_{11\rightarrow10}$	intergenic (+256/+1487)	A1S_0014 → / ← A1S_3477	tyrosyl-tRNA synthetase/hypothetical protein
CP000521	17,656	$(TTCTTTGGA)_{11\rightarrow12}$	intergenic (+264/+1487)	A1S_0014 → / ← A1S_3477	tyrosyl-tRNA synthetase/hypothetical protein
CP000521	17,656	$(TTCTTTGGA)_{11\rightarrow13}$	intergenic (+264/+1487)	A1S_0014 → / ← A1S_3477	tyrosyl-tRNA synthetase/hypothetical protein
CP000521	17,656	$(TTCTTTGGA)_{11\rightarrow16}$	intergenic (+264/+1487)	A1S_0014 → / ← A1S_3477	tyrosyl-tRNA synthetase/hypothetical protein
CP000521	17,656	$(TTCTTTGGA)_{11\rightarrow17}$	intergenic (+264/+1487)	A1S_0014 → / ← A1S_3477	tyrosyl-tRNA synthetase/hypothetical protein
CP000521	17,656	$(TTCTTTGGA)_{11\rightarrow18}$	intergenic (+264/+1487)	A1S_0014 → / ← A1S_3477	tyrosyl-tRNA synthetase/hypothetical protein
CP000521	17,656	$(TTCTTTGGA)_{11\rightarrow19}$	intergenic (+264/+1487)	A1S_0014 → / ← A1S_3477	tyrosyl-tRNA synthetase/hypothetical protein
CP000521	101,403	A→G	intergenic (-614/-2418)	A1S_0081 ← / → A1S_0086	putative signal peptide/hypothetical protein
CP000521	101,415	T→C	intergenic (-626/-2406)	A1S_0081 ← / → A1S_0086	putative signal peptide/hypothetical protein
CP000521	149,238	Δ122 bp		[trnG]	[trnG]
CP000521	485,616	C→T	A375T (GCA→ACA)	A1S_0446 ←	putative transport protein (CPA2 family)
CP000521	486,124	C→T	M205I (ATG→ATA)	A1S_0446 ←	putative transport protein (CPA2 family)
CP000521	511,070	$(ATTTTGAAC)_{2\rightarrow3}$	coding (168/1698 nt)	A1S_0472 ←	2-isopropylmalate synthase
CP000521	516,966	Δ4 bp	coding (252-255/606 nt)	A1S_0476 →	ATP-dependent Clp protease proteolytic subunit
CP000521	517,105	Δ1 bp	coding (391/606 nt)	A1S_0476 →	ATP-dependent Clp protease proteolytic subunit
CP000521	826,432	Δ19 bp	coding (315-333/1227 nt)	A1S_0694 →	FilE
CP000521	826,432	Δ10 bp	coding (315-324/1227 nt)	A1S_0694 →	FilE
CP000521	826,432	Δ1 bp	coding (315/1227 nt)	A1S_0694 →	FilE
CP000521	826,440	Δ2 bp	coding (323-324/1227 nt)	A1S_0694 →	FilE
CP000521	826,441	Δ1 bp	coding (324/1227 nt)	A1S_0694 →	FilE
CP000521	826,444	Δ3 bp	coding (327-329/1227 nt)	A1S_0694 →	FilE
CP000521	826,448	Δ2 bp	coding (331-332/1227 nt)	A1S_0694 →	FilE
CP000521	877,534	2 bp→CA	coding (2165-2166/2940 nt)	A1S_0742 →	iron-regulated protein
CP000521	877,535	T→A	I722I (ATT→ATA)	A1S_0742 →	iron-regulated protein
CP000521	877,895	G→A	L842L (CTG→CTA)	A1S_0742 →	iron-regulated protein
CP000521	877,958	A→T	G863G (GGA→GGT)	A1S_0742 →	iron-regulated protein
CP000521	880,449	C→T	G575D (GGC→GAC)	A1S_0745 ←	hypothetical protein
CP000521	1,029,854	$(TGGCGA)_{2\rightarrow3}$	coding (743/1419 nt)	A1S_0887 →	phosphomannomutase
CP000521	1,030,421	T→C	V437A (GTA→GCA)	A1S_0887 →	phosphomannomutase
CP000521	1,075,007	T→G	intergenic (-102/+7)	A1S_0924 ← / ← A1S_0925	choline dehydrogenase/choline dehydrogenase
CP000521	1,075,007	2 bp→GT	intergenic (-102/+6)	A1S_0924 ← / ← A1S_0925	choline dehydrogenase/choline dehydrogenase
CP000521	1,075,008	G→T	intergenic (-103/+6)	A1S_0924 ← / ← A1S_0925	choline dehydrogenase/choline dehydrogenase
CP000521	1,075,010	C→G	intergenic (-105/+4)	A1S_0924 ← / ← A1S_0925	choline dehydrogenase/choline dehydrogenase
CP000521	1,075,010	2 bp→GC	intergenic (-105/+3)	A1S_0924 ← / ← A1S_0925	choline dehydrogenase/choline dehydrogenase
CP000521	1,075,011	G→C	intergenic (-106/+3)	A1S_0924 ← / ← A1S_0925	choline dehydrogenase/choline dehydrogenase
CP000521	1,217,951	C→T	Q269* (CAA→TAA)	A1S_1055 →	hypothetical protein
CP000521	1,318,577	G→T	V96V (GTC→GTA)	A1S_1127 ←	oxidoreductase short chain dehydrogenase/reductase family
CP000521	1,353,821	G→A	G84S (GGT→AGT)	A1S_1158 →	putative signal peptide
CP000521	1,386,215	$(T)_{8\rightarrow9}$	intergenic (-499/-12)	A1S_1182 ← / → A1S_1183	cyclic AMP receptor protein/hypothetical protein
CP000521	1,630,443	C→T	pseudogene (378/706 nt)	A1S_3662 →	hypothetical protein
CP000521	1,762,595	T→C	D174D (GAT→GAC)	A1S_1513 →	putative membrane protein
CP000521	1,885,761	C→T	T232I (ACT→ATT)	A1S_1616 →	hypothetical protein
CP000521	2,193,897	G→A	D173D (GAC→GAT)	A1S_1893 ←	3-oxoadipate CoA-transferase subunit B
CP000521	2,212,578	C→T	G405D (GGT→GAT)	A1S_1910 ←	ATP-binding protease component
CP000521	2,213,307	G→T	S162* (TCA→TAA)	A1S_1910 ←	ATP-binding protease component
CP000521	2,292,757	+122 bp	intergenic (+265/-11)	A1S_1978 → / → A1S_1979	response regulator protein/putative transcriptional regulator
CP000521	2,374,353	A→G	intergenic (+94/-109)	A1S_3775 → / → A1S_2038	hypothetical protein/hypothetical protein
CP000521	2,396,955	$(GGTTCA)_{17\rightarrow19}$	coding (1230/2109 nt)	A1S_2056 ←	DNA polymerase III tau and gamma subunits (DNA e longation factor III)
CP000521	2,422,982	C→T	intergenic (-34/+290)	A1S_2076 ← / ← A1S_2077	putative outer membrane porin receptor for Fe(II I)-coprogen, Fe(III)-ferrioxamine B and Fe(III)-rhodotrul i c acid uptake (fhuE)/putative outer membrane porin receptor for Fe(II I)-coprogen, Fe(III)-ferrioxamine B and Fe(III)-rhodotrul i c acid uptake (fhuE)
CP000521	2,466,306	G→T	Q41K (CAA→AAA)	A1S_3785 ←	hypothetical protein
CP000521	2,576,310	T→C	E175G (GAG→GGG)	A1S_2213 ←	CsuE
CP000521	2,576,373	A→G	L154S (TTA→TCA)	A1S_2213 ←	CsuE
CP000521	2,576,530	T→C	S102G (AGT→GGT)	A1S_2213 ←	CsuE
CP000521	2,576,533	C→A	D101Y (GAC→TAC)	A1S_2213 ←	CsuE
CP000521	2,576,538	A→G	I99T (ATT→ACT)	A1S_2213 ←	CsuE
CP000521	2,576,586	G→A	S83L (TCG→TTG)	A1S_2213 ←	CsuE
CP000521	2,576,605	A→T	Y77N (TAT→AAT)	A1S_2213 ←	CsuE
CP000521	2,708,492	T→C	L817S (TTA→TCA)	A1S_2337 →	sensory box protein
CP000521	2,708,717	T→A	M892K (ATG→AAG)	A1S_2337 →	sensory box protein
CP000521	2,708,737	A→G	M899V (ATG→GTG)	A1S_2337 →	sensory box protein
CP000521	3,059,254	G→A	Q168* (CAA→TAA)	A1S_2642 ←	regulatory protein TetR
CP000521	3,059,463	C→A	C98F (TGT→TTT)	A1S_2642 ←	regulatory protein TetR
CP000521	3,093,102	G→A	pseudogene (398/1836 nt)	A1S_2668 →	phosphoenolpyruvate carboxykinase
CP000521	3,093,890	G→A	pseudogene (1186/1836 nt)	A1S_2668 →	phosphoenolpyruvate carboxykinase
CP000521	3,129,237	A→G	G15G (GGT→GGC)	A1S_2696 ←	hypothetical protein
CP000521	3,306,773	$(T)_{8\rightarrow7}$	coding (304/1212 nt)	A1S_2858 ←	putative methyltransferase
CP000521	3,328,022	T→C	V168A (GTC→GCC)	A1S_2878 →	hypothetical protein
CP000521	3,434,070	Δ2 bp	coding (886-887/1194 nt)Icoding (4-5/105 nt)	A1S_3863 →<br>A1S_3862 ←	hypothetical protein<br>hypothetical protein
CP000521	3,441,396	Δ1 bp	intergenic (-28/+371)	A1S_r12 ← / ← A1S_2955	16S ribosomal RNA/peptidyl prolyl-cis-trans-isomerase rotamase
CP000521	3,468,548	$(ATGGTG)_{9\rightarrow10}$	coding (454/987 nt)<br>coding (193/231 nt)	A1S_2977 ←<br>A1S_3873 →	cation diffusion facilitator family transporter<br>hypothetical protein
CP000521	3,468,548	$(ATGGTG)_{9\rightarrow11}$	coding (454/987 nt)<br>coding (193/231 nt)	A1S_2977 ←<br>A1S_3873 →	cation diffusion facilitator family transporter<br>hypothetical protein
CP000521	3,500,412	T→C	intergenic (-119/+6)	A1S_3014 ← / ← A1S_r13	hypothetical protein/5S ribosomal RNA
CP000521	3,500,576	G→A	intergenic (-25/+140)	A1S_r13 ← / ← A1S_r14	5S ribosomal RNA/23S ribosomal RNA
CP000521	3,500,617	Δ1 bp	intergenic (-66/+99)	A1S_r13 ← / ← A1S_r14	5S ribosomal RNA/23S ribosomal RNA
CP000521	3,500,634	C→T	intergenic (-83/+82)	A1S_r13 ← / ← A1S_r14	5S ribosomal RNA/23S ribosomal RNA
CP000521	3,870,029	Δ21 bp	pseudogene (577-597/605 nt)	A1S_3363 ←	membrane metalloendopeptidase protein
CP000521	3,870,072	$(CTCCAGATG)_{5\rightarrow6}$	pseudogene (554/605 nt)	A1S_3363 ←	membrane metalloendopeptidase protein
CP000521	3,870,072	$(CTCCAGATG)_{5\rightarrow13}$	pseudogene (554/605 nt)	A1S_3363 ←	membrane metalloendopeptidase protein
CP000521	3,928,225	$(G)_{7\rightarrow6}$	coding (28/780 nt)	A1S_3417 →	regulatory proteins IclR
CP000521	3,928,225	$(G)_{7\rightarrow8}$	coding (28/780 nt)	A1S_3417 →	regulatory proteins IclR
CP000521	3,962,192	T→C	L393P (CTG→CCG)	A1S_3449 →	phosphoenolpyruvate carboxylase
